# Supplementary material for: Margin between success and failure of PDA stenting for duct-dependent pulmonary circulation
Source: PLoS One. 2022 Apr 14;17(4):e0265031. doi: 10.1371/journal.pone.0265031 (PMC9009684; doi:10.1371/journal.pone.0265031)
Supplement: Attachment — Submitted filename: Rebuttal Letter PDA stent.docx [file pone.0265031.s001.docx]

**Rebuttal Letter**

The authors thank the editors and the reviewers for their valuable comments, this letter includes a response to the individual remarks

**General Comments**

1. The title was adjusted to “Margin between success and failure of PDA stenting for duct-dependent pulmonary circulation” according to the journal’s requirements
2. Data availability and minimal data set: The main Excel is added as supplement
3. Tables included in the main manuscript
4. Figure legends added at end of manuscript
5. Authorship: the author (Baher Hanna) and (Mai Hussein) that were missed in the initial manuscript were re-added. And their contribution to the manuscript was added.
6. Definition of a successful procedure was added under “patients and methods”

**Reviewer One:**

- Latest evidences are not in favor of PDA stenting in univentricular heart. Thus inclusion criteria should be well discussed. Is a policy of author's center perform so many PDA stenting. How the Center's volume?

We added 3 extra references (25-28) that compare PDA stents to surgical shunts that tend to favor PDA stenting. Our institution performs around 900 catheterization cases per year: on average 50 of them are PDA stenting. Over the past 3 years, the small sized 3 and 3.5mm surgical shunts have been very sparse in the country and we resolve to PDA stenting as a life-saving procedure even in some cases in which a BT shunt would have been the choice if available.

- Introduction and Discussion: discuss critically the advantages/disadvantages of PDA stenting, according to current literature

The first paragraph under “Discussion” was edited as such

- Also in the abstract a short summary of global success/complication rate is missing

Added to the abstract

- Results: data should be discussed first all together and then divided into subgroups

The section “Results” was edited accordingly

- Is there a surgical group for comparison?

No, the aim of our study was to evaluate the different approaches for PDA stenting and the parameters reflecting the outcome in this specific cohort, rather than comparing results to surgical shunts. As mentioned earlier, in our country we are commonly obliged to perform a life-saving intervention due to the unavailability of small sized BT shunts.

- Limitations and discussion: Results are in line with previous publication, but complication rate is high. Why to prefer PDA stent to m-BT shunt?

In our institution, complications of neonatal MBTS are higher than PDA stents. A study to compare both is yet to be done.

- Limitations section: should be much more robust. Patient's selection criteria is a clear limitation. The choice of the material seems also to be quite arbitrary

The “Limitations” section was edited accordingly

**Reviewer Two:**

- The concept and the main topic of this article (proper vascular access depending upon duct morphology) is not new and it has been extensively reported.

We are mainly concerned with the indicators of a successful procedure based on the ductal morphology, origin and approach

- Detailed information regarding the sub-group of patients with failed PDA stenting (18 patients, 19%) should be provided, such as specific reason for failure

Results have been added to contain detailed information on the subgroups, details for procedural failure are discussed under “results” and Supplement Tables
- There is a concerning high rate of complication (including vascular and stent displacement), this should be discussed and explained
This was mentioned in “results” The main cause of procedural failure was inadequate parked coronary wire inside one of the branch of pulmonary arteries (14 cases; 77.7%), while 2 cases (11.1%) were complicated by acute stent thrombosis, and another 2 cases with stent dislodgment. Other procedural complications comprised femoral artery thrombosis in 7 cases (7.2%), No patients died on table or within 6 hours of the procedure.

- There is no specific protocol described regarding the institutional approach to duct-dependent lesion. More specifically, is every patient with duct-dependant circulation referred to your institution considered for PDA stent, irrespective of other parameters? Do you select patient referred for transcatether intervention?
In our institution, we are frequently obliged to perform life-saving PDA stenting because of the unavailability of small sized surgical shunts in the country. This was mentioned under “limitations”.

- Do you have a surgical comparisons group undergone BT shunt during the same period. Long-term comparison of such groups would be of some interest
No we currently do not, such a study is yet to be done.

- Do you have any follow-up surgical data at the time of corrective surgery regarding this patient population?

Fifty-eight patients (74%) survived to hospital discharge after a mean period of 4 days (3-9 days). A total of 43 patients were followed-up for a period of 3-12 months: whereas 10 patients died; and 5 patients dropped follow-up. Oxygen saturation ranged from 79-90% showing gradual decline over time (figure 2). MDCT angiography was performed whenever oxygen saturation falls beneath 75%, or prior to planned surgical intervention. In our cohort, this imaging modality was done at a mean of 4 months post-procedure and showed significant growth of pulmonary arteries (Table 3). Surgical palliation and repair: 9 cases underwent partial cavo pulmonary connection (PCPC), 6 cases underwent Modified Blalock Taussig Thomas shunt and 3 cases underwent biventricular repair (Figure 3).

- Did you ever change vascular access after baseline angiography? In other words, were you always able to correctly identify PDA morphology and establish the correct vascular approach using pre-procedural echocardiography?
It is our routine to perform an initial aortogram through the femoral artery, accordingly to which the approach is decided. In 2 cases the approach was switched during the procedure. This is mentioned under “results”.

Based on ductal origin and morphology, the approach to ductal stenting was femoral artery in 72 patients (75%); axillary artery in 19 patients (19.8%), carotid artery in 3 patients (3.1%), and femoral vein in 2 patients (2.1%). It is of note that 2 cases that were initiated for femoral artery approach then shifted to the axillary route

- There are significant procedural data missing: type, timing and dosing of anticoagulation, description of angiography, type of wire used to deploy stent.
These data were added under “patients and methods”

**Procedures:** The femoral arterial access was obtained in all patients by 4 French short sheath, 100 units/kg unfractionated intravenous heparin and prophylactic antibiotic was given to every patient followed by aortogram using 4 French pigtail catheter. Patients were divided into 4 groups according to PDA origin: Group 1 with PDA from proximal descending aorta, Group 2 with PDA from undersurface of aortic arch, Group 3 with PDA arising opposite the subclavian artery and Group 4 with PDA arising opposite the innominate/brachiocephalic artery [figure1A-H]. Based on this angiogram data, the length of the stent and its diameter was selected as well as the best access route, i.e. whether to proceed with the femoral arterial access or to convert to another approach like axillary, carotid or femoral vein access. Two different types of catheters either 4 French cut pigtail or 4 French right Judkin [JR]were used for [encroaching](https://www.google.com/search?sxsrf=ALeKk03rTXqlvgEY9Yq6g-1fSLM5lgv1Zw:1602862852414&q=encroaching+meaning+in+arabic&spell=1&sa=X&ved=2ahUKEwiU_MO3ubnsAhXiz4UKHeLXAysQkeECKAB6BAgDECk) of PDA. PDA was crossed with 0.014 inch percutaneous transluminal coronary angioplasty [PTCA] wire [Middle Support Weight guide wire, Boston Scientific, USA] and it was parked in either of the branch pulmonary arteries . In all cases, pre-mounted bare metal stents [architect, Life Vascular Device Biotech, Barcelona, Spain] or drug-eluting coronary stents [Monorail, Everolimus-Eluting Platinum Chromium, Boston Scientific, USA]. The diameter was chosen with respect to the weight of the patient, size of the PDA, its constricted segment and branch pulmonary arteries. Length of stent was chosen to cover the entire length of the duct with proximal end into aorta and distal end into branch PA..The selected coronary stent was tracked over PTCA wire and placed across the PDA with its proximal end in the aorta and distal end in branch pulmonary artery. After confirming the position of the stent, it was deployed with its initial nominal pressure then re-dilated for better apposition. Balloon was removed after deployment keeping the wire in situ. Injection was done again to check the position of the stent, flow across the stent and branch pulmonary arteries. After achieving satisfactory results, wire was removed. In cases of branch pulmonary artery origin stenosis, we aimed to deploy the stent distal to the stenosed segment. After stent implantation, angiogram injection was performed to confirm the stent position. All the patients were shifted to ICU and received  unfractionated heparin  infusion on 20 IU/kg/hr for 24-48 hours (partial prothrombin time was maintained 2–3 times of normal). All the patients were given antiplatelets in form of aspirin on 5mg/kg/day that was continued until undergoing second-stage palliation or repair. Procedural success of PDA stenting was defined as the implantation of a well-seated stent, good flow for both pulmonary arteries and stable SpO2 above 75% for a hemodynamic-stable patient.

- Long term follow up data are essential
Our study’s end-point was the 2^nd^ surgical intervention; either the cavo-pulmonary shunt for univentricular heart or biventricular repair as definitive surgery.
