# Supplementary material for: Margin between success and failure of PDA stenting for duct-dependent pulmonary circulation
Source: PLoS One. 2022 Apr 14;17(4):e0265031. doi: 10.1371/journal.pone.0265031 (PMC9009684; doi:10.1371/journal.pone.0265031)
Supplement: Attachment — Submitted filename: rebuttal letter-2.docx [file pone.0265031.s003.docx]

**Rebuttal Letter**

The authors thank the editors and the reviewers for their valuable comments, this letter includes a response to the individual remarks

1. If the authors have adequately addressed your comments raised in a previous round of review and you feel that this manuscript is now acceptable for publication, you may indicate that here to bypass the “Comments to the Author” section, enter your conflict of interest statement in the “Confidential to Editor” section, and submit your "Accept" recommendation.

Reviewer #3: (No Response)

Reviewer #4: All comments have been addressed

Reviewer #5: (No Response)

2. Is the manuscript technically sound, and do the data support the conclusions?

The manuscript must describe a technically sound piece of scientific research with data that supports the conclusions. Experiments must have been conducted rigorously, with appropriate controls, replication, and sample sizes. The conclusions must be drawn appropriately based on the data presented.

Reviewer #3: Yes

Reviewer #4: Yes

Reviewer #5: Yes

3. Has the statistical analysis been performed appropriately and rigorously?

Reviewer #3: I Don't Know

Reviewer #4: Yes

Reviewer #5: Yes

4. Have the authors made all data underlying the findings in their manuscript fully available?

The [PLOS Data policy](http://www.plosone.org/static/policies.action#sharing) requires authors to make all data underlying the findings described in their manuscript fully available without restriction, with rare exception (please refer to the Data Availability Statement in the manuscript PDF file). The data should be provided as part of the manuscript or its supporting information, or deposited to a public repository. For example, in addition to summary statistics, the data points behind means, medians and variance measures should be available. If there are restrictions on publicly sharing data—e.g. participant privacy or use of data from a third party—those must be specified.

Reviewer #3: Yes

Reviewer #4: Yes

Reviewer #5: Yes

5. Is the manuscript presented in an intelligible fashion and written in standard English?

PLOS ONE does not copyedit accepted manuscripts, so the language in submitted articles must be clear, correct, and unambiguous. Any typographical or grammatical errors should be corrected at revision, so please note any specific errors here.

Reviewer #3: Yes

Reviewer #4: Yes

Reviewer #5: No

The manuscript has been revised and the language edited

6. Review Comments to the Author

Please use the space provided to explain your answers to the questions above. You may also include additional comments for the author, including concerns about dual publication, research ethics, or publication ethics. (Please upload your review as an attachment if it exceeds 20,000 characters)

Reviewer #3: My previous comments on this manuscript are not addressed in the response by the authors, so I have no further comments at this time.

Reviewer #4: The authors answered my questions and the paper has a huge impact on the field. I strongly advise to publish

Reviewer #5: I am not an expert at the first-version review, so I only comment on whether the author's answer to the question from the expert at the first-version review is adequate.
1) The purpose of the research: The abstract states that it is necessary to analyze the factors of the success of the operation. In fact, it is grouped and compared according to the origin of the PDA. In the following discussion, it is said that this study mainly wants to analyze the situation of different approaches and influence the success of the operation. The indicators that the author ultimately wants to be clear need to be consistent. If the main research in the discussion is the influence of different approaches on the success rate，why not group them using approaches?

The approach to PDA stenting is directly related to its origin, this has been clarified in the final edition
2) In the method part, the second reviewer asked the author to give a detailed description of the inclusion and exclusion criteria. The author’s answer was not very detailed. Although there are intermittent descriptions in the method part of the article, such as weight, coagulation dysfunction, etc., it should be systematically Write down the inclusion and exclusion criteria for patients who actually undergo PDA stent placement; this is also a question raised by the reviewer in the limitation section.

As indicated in the “material and methods” section (lines 1-3), all cases presenting for ductal stenting during the 2 year study period were included, only excluding those with untreated sepsis or coagulopathy.

3) The results part: there are overlaps description in results. The first two paragraphs are for the explanation of the patient's baseline data. There is no need to write approach groupings. Is it better to describe in the procedure part?

Done

And data in the table does not need to be described all them again. It is possible to group by PDA origin, but in fact, according to the author's results, the procedure approach, PDA distortion, and complications of groups 2-4 are similar, and can be used as a unified group for comparison with the first group.

The authors believe that grouping the PDA according to its origin would be more informative to the interventionist who is planning for the procedure, therefore needs to decode on the best approach based on the PDA anatomy.

Regarding the follow-up part, 43 patients were followed up for 3-12 months. How many cases were actually followed up for 4 months? What is the actual number of people undergoing MDCT angiography 4 months after surgery? This is not described in detail. “The detailed information regarding the sub-group of patients with failed PDA stenting should be provided ”which was proposed by the second reviewer, was not discussed adequately.

All cases were assessed routinely by MDCT angiography prior to Glenn, but not after the operation. This was adjusted in the results.

7. PLOS authors have the option to publish the peer review history of their article ([what does this mean?](https://journals.plos.org/plosone/s/editorial-and-peer-review-process#loc-peer-review-history)). If published, this will include your full peer review and any attached files.
If you choose “no”, your identity will remain anonymous but your review may still be made public.
**Do you want your identity to be public for this peer review?** For information about this choice, including consent withdrawal, please see our [Privacy Policy](https://www.plos.org/privacy-policy).

Reviewer #3: No

Reviewer #4: **Yes:** Shai Tejman Yarden MD MSc MBA

Reviewer #5: No
