# Supplementary material for: Margin between success and failure of PDA stenting for duct-dependent pulmonary circulation
Source: PLoS One. 2022 Apr 14;17(4):e0265031. doi: 10.1371/journal.pone.0265031 (PMC9009684; doi:10.1371/journal.pone.0265031)
Supplement: Attachment — Submitted filename: New Microsoft Word Document.docx [file pone.0265031.s004.docx]

**Rebuttal Letter**

The authors thank the editors and the reviewers for their valuable comments, this letter includes a response to the individual remarks

1. If the authors have adequately addressed your comments raised in a previous round of review and you feel that this manuscript is now acceptable for publication, you may indicate that here to bypass the “Comments to the Author” section, enter your conflict of interest statement in the “Confidential to Editor” section, and submit your "Accept" recommendation.

Reviewer #3: (No Response)

Reviewer #5: All comments have been addressed

2. Is the manuscript technically sound, and do the data support the conclusions?

The manuscript must describe a technically sound piece of scientific research with data that supports the conclusions. Experiments must have been conducted rigorously, with appropriate controls, replication, and sample sizes. The conclusions must be drawn appropriately based on the data presented.

Reviewer #3: Yes

Reviewer #5: Yes

3. Has the statistical analysis been performed appropriately and rigorously?

Reviewer #3: No

Reviewer #5: Yes

4. Have the authors made all data underlying the findings in their manuscript fully available?

The [PLOS Data policy](http://www.plosone.org/static/policies.action#sharing) requires authors to make all data underlying the findings described in their manuscript fully available without restriction, with rare exception (please refer to the Data Availability Statement in the manuscript PDF file). The data should be provided as part of the manuscript or its supporting information, or deposited to a public repository. For example, in addition to summary statistics, the data points behind means, medians and variance measures should be available. If there are restrictions on publicly sharing data—e.g. participant privacy or use of data from a third party—those must be specified.

Reviewer #3: Yes

Reviewer #5: Yes

5. Is the manuscript presented in an intelligible fashion and written in standard English?

PLOS ONE does not copyedit accepted manuscripts, so the language in submitted articles must be clear, correct, and unambiguous. Any typographical or grammatical errors should be corrected at revision, so please note any specific errors here.

Reviewer #3: Yes

Reviewer #5: Yes

6. Review Comments to the Author

Please use the space provided to explain your answers to the questions above. You may also include additional comments for the author, including concerns about dual publication, research ethics, or publication ethics. (Please upload your review as an attachment if it exceeds 20,000 characters)

Reviewer #3: This is the second revision I have reviewed after I sent comments for revision. Incredibly, the authors have still not addressed my suggestions. For example, References #25 and 28 are still identical. The fact that "Group 4" only contains 1 member and yet is dealt with statistically, is not acceptable.
I have nothing further to say, and will not render any other decisions. I WILL NOT REVIEW THIS MANUSCRIPT AGAIN.

The changes made are:

- group 4: indicated that it was not included in statistical analysis being a single case, the corresponding column from table 1 has been deleted

- reference number 28 has been deleted and the numbering within the text adjusted

Reviewer #5: All questions mentioned in previous review have received more detailed and serious responses.The writing of the article has also been thoroughly improved.

7. PLOS authors have the option to publish the peer review history of their article ([what does this mean?](https://journals.plos.org/plosone/s/editorial-and-peer-review-process#loc-peer-review-history)). If published, this will include your full peer review and any attached files.


If you choose “no”, your identity will remain anonymous but your review may still be made public.


**Do you want your identity to be public for this peer review?** For information about this choice, including consent withdrawal, please see our [Privacy Policy](https://www.plos.org/privacy-policy).

Reviewer #3: No

Reviewer #5: No
