# Supplementary material for: Margin between success and failure of PDA stenting for duct-dependent pulmonary circulation
Source: PLoS One. 2022 Apr 14;17(4):e0265031. doi: 10.1371/journal.pone.0265031 (PMC9009684; doi:10.1371/journal.pone.0265031)
Supplement: Attachment — Submitted filename: new Rebuttal 2.docx [file pone.0265031.s005.docx]

**Rebuttal letter**

The authors thank the editors and the reviewers for their valuable comments, this letter includes a response to the individual remarks

1. If the authors have adequately addressed your comments raised in a previous round of review and you feel that this manuscript is now acceptable for publication, you may indicate that here to bypass the “Comments to the Author” section, enter your conflict of interest statement in the “Confidential to Editor” section, and submit your "Accept" recommendation.

Reviewer #2: (No Response)

Reviewer #5: All comments have been addressed

Response: Thank you for your kind response

2. Is the manuscript technically sound, and do the data support the conclusions?

The manuscript must describe a technically sound piece of scientific research with data that supports the conclusions. Experiments must have been conducted rigorously, with appropriate controls, replication, and sample sizes. The conclusions must be drawn appropriately based on the data presented.

Reviewer #2: No

Reviewer #5: Yes

Response: Thank you for your kind response. The conclusion was based upon the supported data and results of statistical significance

3. Has the statistical analysis been performed appropriately and rigorously?

Reviewer #2: No

Reviewer #5: Yes

Response: Thank you for your kind response .

Numerical data were expressed as median and interquartile range [25th -75th percentiles] while categorical data were expressed as numbers or numbers and percentages. Comparisons between groups were calculated using non-parametric Kruskal Wallis, Mann-Whitney and Wilcoxon signed-rank tests for numerical data, while χ2 tests were used for comparison between groups for categorical data. Logistic regression was performed for evaluation of outcome predictors. Statistical significance was considered if P-values <0.05.

4. Have the authors made all data underlying the findings in their manuscript fully available?

The PLOS Data policy requires authors to make all data underlying the findings described in their manuscript fully available without restriction, with rare exception (please refer to the Data Availability Statement in the manuscript PDF file). The data should be provided as part of the manuscript or its supporting information, or deposited to a public repository. For example, in addition to summary statistics, the data points behind means, medians and variance measures should be available. If there are restrictions on publicly sharing data—e.g. participant privacy or use of data from a third party—those must be specified.

Reviewer #2: Yes

Reviewer #5: Yes

Response: Thank you for your kind response

5. Is the manuscript presented in an intelligible fashion and written in standard English?

PLOS ONE does not copyedit accepted manuscripts, so the language in submitted articles must be clear, correct, and unambiguous. Any typographical or grammatical errors should be corrected at revision, so please note any specific errors here.

Reviewer #2: Yes

Reviewer #5: Yes

Response: Thank your for you kind response

6. Review Comments to the Author

Please use the space provided to explain your answers to the questions above. You may also include additional comments for the author, including concerns about dual publication, research ethics, or publication ethics. (Please upload your review as an attachment if it exceeds 20,000 characters)

Reviewer #2: This study reports on the use of PDA stenting in the setting of resource-constrained country where availabiity of surgica shunt is limited. Although of some interest this reviewer think that this study is not adding significant novelty to the field.

Reviewer #5: All questions mentioned in previous review have received more detailed responses. No more suggestions.

Response: Thank you for your kind response. Priority for ductal stenting will be in countries with limited resources, long waiting surgical lists and unavailability of small B-T shunt so the authors think about the treatment of duct dependent pulmonary circulation and different factors that predict the outcome of this procedure.

7. PLOS authors have the option to publish the peer review history of their article (what does this mean?). If published, this will include your full peer review and any attached files.

If you choose “no”, your identity will remain anonymous but your review may still be made public.

Do you want your identity to be public for this peer review? For information about this choice, including consent withdrawal, please see our Privacy Policy.

Reviewer #2: No

Reviewer #5: No
